# Nanomaterial-Based Sensors for Coumarin Detection

**DOI:** 10.1021/acsomega.4c01945

**Published:** 2024-07-05

**Authors:** Yeşeren Saylan, Nilufer Aliyeva, Seckin Eroglu, Adil Denizli

**Affiliations:** 1 Department of Chemistry, Hacettepe University, 06800 Ankara, Turkey; 2 Department of Biological Sciences, Middle East Technical University, 06800 Ankara, Turkey

## Abstract

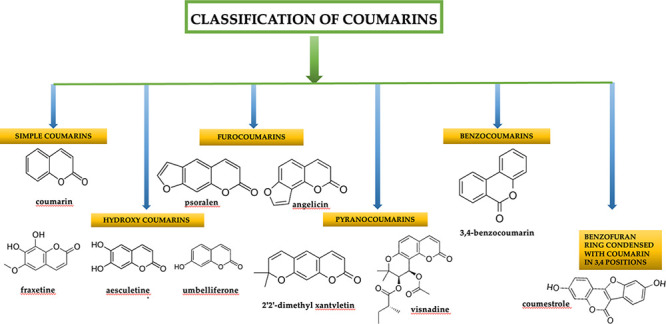

Sensors are widely used owing to their advantages including excellent sensing performance, user-friendliness, portability, rapid response, high sensitivity, and specificity. Sensor technologies have been expanded rapidly in recent years to offer many applications in medicine, pharmaceuticals, the environment, food safety, and national security. Various nanomaterial-based sensors have been developed for their exciting features, such as a powerful absorption band in the visible region, excellent electrical conductivity, and good mechanical properties. Natural and synthetic coumarin derivatives are attracting attention in the development of functional polymers and polymeric networks for their unique biological, optical, and photochemical properties. They are the most abundant organic molecules in medicine because of their biological and pharmacological impacts. Furthermore, coumarin derivatives can modulate signaling pathways that affect various cellular processes. This review covers the discovery of coumarins and their derivatives, the integration of nanomaterial-based sensors, and recent advances in nanomaterial-based sensing for coumarins. This review also explains how sensors work, their types, their pros and cons, and sensor studies for coumarin detection in recent years.

## Introduction

Nanotechnology is a science dealing with the production of nanoparticles whose sizes vary from 1 to 100 nm, as well as the modification of particle structure and size using various synthesis strategies.^1^ Nanotechnology is a complex interdisciplinary field that includes nanoscience, nanomaterials, nanochemistry, nanophysics, nanoelectronics, and nanobionics. Nanomaterials provide essential tools for the facile engineering and fine-tuning of unequaled sensing configurations depending on recognition events at the nanoscale.^2^ For this reason, many nanomaterials are commonly used in sensor design in various platform areas.^3^ Various nanomaterials such as nanoparticles, nanotubes, nanofibers, nanowires, nanorods, nanocomposites, nanopolymers, nanofilms, and nanoplates are used.^4^ Numerous nanomaterial-based sensors have been developed for many applications using carbon nanotubes, gold nanomaterials, graphene, nanomotors, nanocables,^5^ carbon spherical shells,^6,7^ and carbon nanoparticles.^8−11^ While graphene is a rising star among nanomaterials, carbon nanotubes have remained stable. Gold nanoparticles grow moderately, and nanomotors fall below the proof of principle level.^4^ Analytical methods based on nanomaterials offer numerous possibilities for the development of devices and sensors with remarkable physicochemical properties, high surface/volume ratios, and high reactive and adsorption capacities, as well as fine-tuning the required sensor configuration at the nanoscale for risk assessment of environmental contaminants.^12^ Sensors made using nanomaterials have been seen to have advantages such as portability, miniaturization, and rapid analysis.^13^ Sensors are affected by the heat, light, humidity, motion, pressure, and many other environmental events they detect. They are exploited in industry^14^ and our daily lives^15^ to detect a range of sources, including light, temperature,^16^ pressure, voltage, thermal energy,^17^ and strain.^18,19^ After measuring a physical feature, sensors record, respond to, or display it differently.^20^ Sensors are devices that sense changes in the environment, gather signals, and work out answers regarding them.^21^ Various sensors are exploited daily to make more valid and faster analyses.^22−28^ Apart from these, sensors can provide sensitivity and selectivity. They are analytical tools that can be miniaturized and automated.^29^ The name “coumarin” comes from the French term “Coumarou”, referring to the tonka bean from which Vogel first isolated coumarin in 1820.^30,31^ Afterward, it was discovered in several other plants, including cinnamon, vanilla grass, sweet clover, strawberries, currants, apricots, etc.^32^ In nature, coumarin and its derivatives occur freely or in combination with other molecules, such as glycosides.^33^ Plant coumarins are produced through the phenylalanine, shikimic acid, and cinnamic acid pathways.^34^ Coumarins make up a family of secondary metabolites that start from phenylpropane. They are organic heterocyclic compounds. Coumarin and its derivatives have a benzene ring appended to an α-pyrone ring in their main structure.

This Review encompasses the exploration of coumarins and their derivatives, the incorporation of sensors based on nanomaterials, and the latest developments in sensing coumarins using nanomaterial-based approaches. Additionally, the Review comprehensively examines the mechanisms, various types, advantages, and disadvantages of sensors and recent studies focusing on coumarin detection performed in the past few years.

## Coumarin

### Coumarin and Coumarin Derivatives

Coumarin (2*H*-benzopyran-2-ones) is an ordinary scaffold outspread in nature, many plants, and some fungi and bacteria.^35^ Coumarins have one-of-a-kind features like easy derivatization, wide Stokes shift, low toxicity, elevated fluorescence quantum yield, and excellent photostability.^36^ Coumarin is a pleasant fragrance in products, such as foods, drinks, and tobacco. It is illegally put into food in such a small amount as a spice that it is very hard to detect.^37^ After experiments on animals, it was confirmed that coumarin is carcinogenic, so certain countries have set a safe dose range.^38^ Plants synthesize vast amounts of natural metabolites, named secondary metabolites.^39,40^ Secondary metabolites have essential ecological functions, promoting plant defenses against herbivores and microorganisms and playing a role in luring pollinators. Secondary metabolites are used by humans as medicinal, flavoring, and aromatization substances.^41^ As a result of extensive pharmacological and phytochemical research in the last few decades, more than 400 coumarins have been identified in scientific publications in the past few years. Although natural coumarins are seen in high concentrations in cassia, cinnamon, and tonka beans, they are obtained in small amounts in apricots and strawberries. For example, tonka beans contain 1–3% coumarin.^42^ Studies have shown that even tiny quantities may cause severe liver damage within several weeks.^43^ Plants containing coumarin have a sweet smell but are bitter, so animals should stay away from them.

Depending on their chemical texture, coumarin and its derivatives have various biological properties. Therefore, coumarin has anti-inflammatory, anticoagulant, antimicrobial, antiviral, anticancer, antihypertensive, antituberculous, anticonvulsant, anti-HIV, antiadipogenic, neuroprotective, antihyperglycemic, and antioxidant properties.^44−48^ Many P450 enzymes play a role in the biosynthesis of coumarin, whose structure consists of two six-membered rings and lactone carbonyl groups. O-hydroxylation is an essential step in coumarin biosynthesis in plants. Many coumarins have significant optical activity and thermal stability. Among the approved anticancer drugs, around 80% are derived from natural compounds. Lately, these natural compounds have attracted the interest of scientists thanks to their great variety of biological activities, especially since they can work with various enzymes and receptors such as kinases, monocarboxylate transporters, telomerases, aromatases, sulfatases, and carbonic anhydrases in living organisms. Thus, they have potential activity against several cancer cell lines.^49−51^ Therefore, coumarin is a good model for improving new anticancer agents. Because the coumarin core is easily assembled and decorated, new coumarin-based compounds can be developed, allowing for their potential use in treating diseases such as cancer.^52^

**Figure 1:**
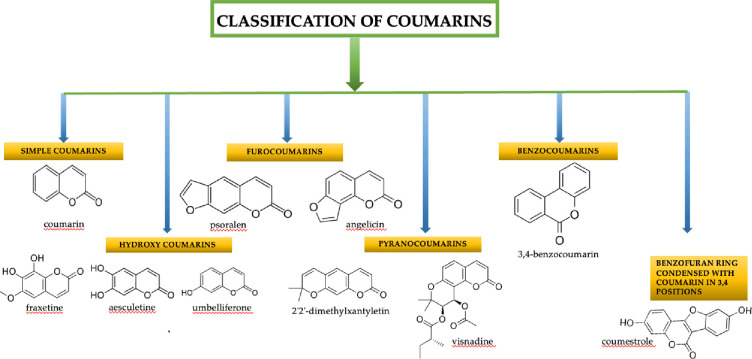
Classification of the main coumarins.

As shown in Figure 1, coumarins are divided into categories based on the various substituents on the benzene ring: simple coumarins, pyranocoumarins, furocoumarins, benzocoumarin, and hydroxycoumarin.^53,54^ Simple coumarins, including 4-hydroxycoumarin, scopoletin, esculetin, and 7-hydroxycoumarin (umbelliferone), are obtained by the catalysis of hydroxide radicals and methyl groups at different positions. They are comprised of the simplest coumarin compounds and their glycosylated, alkylated, hydroxylated, and alkoxylated derivatives. Complex coumarins obtained from plants are formed via the phenylpropanoid pathway.^55^ Coumarin-based derivatives have a phenolic hydroxyl group, produced as one of the most derivative functional groups. The most significant class of 1-benzopyran derivatives is the coumarins.^56^ Coumarin derivatives constitute an important class of natural plant metabolites with diverse biological activities. They can also be obtained synthetically.^57,58^ Like coumarins, their derivatives are also recognized as interesting sources for drug exploration and biological activity improvements. Lately, along with the improvement of herbal medicines, it has been shown that coumarin and its derivatives are used in various platforms, including dyes, insecticides, sensitizers, herbicides, food additives, antioxidant reagents, perfumes, and cosmetics.

Scopoletin, also known as 6-methoxy-7-hydroxycoumarin, is one of the natural coumarins. It is widely present in various edible plants and plays an important role in human health. Structurally, scopoletin has two aromatic rings supported by a hydroxyl group substituent and oxo and methoxy groups.^59^ Recently, further research has been conducted on scopoletin as a functional monomer in electropolymerization in molecularly imprinted polymers intended for sensor use.^60^ Scopoletin is used as a monomer in the synthesis of sensitive polymers due to its advantages, such as easy polymerization, low cost, water solubility, and the ability to use aqueous solutions rather than toxic solvents.^61,62^ Esculetin, also known as 6,7-dihydroxycoumarin, is one of the simplest coumarins. It is an aglycone metabolite of esculin. Esculetin is a naturally occurring dihydroxycoumarin.^63,64^ It is derived mainly from the bark and root bark of the Chinese herb *Fraxinus rhynchophylla*. Hence, it has broad-spectrum pharmacological and antibacterial activity. It is hoped that esculetin will become a therapeutic drug for the treatment of particular diseases like cancer, diabetes, atherosclerosis, Alzheimer’s disease, Parkinson’s disease, and nonalcoholic fatty liver disease. Studies have shown that the oral bioavailability of esculetin is low. Glucuronidation has been identified as the main metabolic pathway of esculetin, and C-7 phenolic hydroxyl was the main metabolic site.^65^ Warfarin is a synthetic derivative of the natural anticoagulant substance dicoumarol. It delays blood clotting by inhibiting the synthesis of vitamin K-based clotting factors in the liver. Warfarin is an oral anticoagulant that is effective in the treatment and prevention of venous thromboembolism (VTE), pulmonary embolism, acute myocardial infarction, prosthetic heart valves, stroke, atrial fibrillation, or peripheral arterial disease.^66,67^ Psoralen and angelicin, isolated from the traditional Chinese medicine *Psoralea*, are widely used in the treatment of bone diseases and immune regulation. Angular furanocoumarins such as angelicin, which have a furan ring attached to positions 7 and 8 of the coumarin ring, cannot bind to DNA due to their geometry and are therefore less phototoxic. Angelicin is related to psoralen, another member of the furanocoumarin group that is known to be effective in phototherapy. Angelicin has demonstrated antitumor properties via intrinsic and extrinsic apoptotic pathways in multiple cell lines. It also has the ability to inhibit tubulin polymerization to a greater extent than psoralen.^68,69^ Catechol, an organic compound with the molecular formula C_6_H_4_(OH)_2_, is also called pyrocatechol or 1,2-dihydroxybenzene.^70^ Catechol, the *ortho*-isomer of three isomeric benzenes, is one of the phenolic compounds that constitute the majority of root exudates in response to iron deficiency in noncereal plants, and there is evidence that it is present in many other soluble root exudates, like coumarin.^71^ Catechols are commonly used in the pesticide, plastic, tanning, dye, cosmetic, and pharmaceutical industries; since it is released into the environment as a source of industrial waste and environmental contamination, it can be toxic to humans and animals even at very low doses.^72,73^ For this reason, it is essential to improve a rapid and effective method for catechol detection.^74,75^

### Coumarin Detection Methods

At present, known techniques for coumarin detection are liquid chromatography–tandem mass spectrometry (LC-MS), high-performance liquid chromatography (HPLC), capillary electrophoresis (CE), enzyme-linked immunosorbent assay (ELISA), gas chromatography (GC), and gas chromatography–mass spectrometry (GC-MS). The most commonly used method is HPLC, which is often paired with a diode array detector or ultraviolet detector.^76^ However, the complexity, time consumption, low sensitivity, and poor reproducibility of these techniques have shown that improving a new method for coumarin detection and its derivatives is essential. In particular, to eliminate the toxic side effects of coumarins, scientists have stepped up work to develop a plan and fast method for determining coumarins.^77−80^

Chromatographic methods are exploited like reference analytical methods for coumarin determination because they provide accurate and automatic results. Nevertheless, the need for costly equipment and reagents, stringent tentative circumstances, long analysis times, labor-intensive analysis, sample pretreatment, slow reaction times, and expert employees restrain their application. Studying and improving analytical methodologies leveraging sensors for environmental purposes can help overcome the mentioned challenges.^81^ Typically, the sensor utilizes a recognition element directly touching the transducer to procure particular, quantitative, or semiquantitative analytical data. According to this description, a sensor usually has a recognition element, a transducer, and an electronic system. The recognition element ensures accurate identification of the analyte in the source matrix. The transducer converts the interaction between an analyte and a recognition element into a measurable signal. Finally, the electronic system serves to strengthen and process the signal. Nanomaterial-based sensors are nanoscale tools that detect specific biological chemical compounds and environmental occurrences.^27,82,83^ These sensors are more economical, specific, and intelligent than their macroscopic counterparts. Generally, the dynamic range, reaction time, selectivity, and LOD determine the reproducibility of such devices. Their work can be increased with the use of nanomaterials. In recent years, many nanomaterials have begun to be used to produce sensors for the determination and quantification of analytes in the environment. Nanomaterial-based sensors can play many main roles: (i) they provide a larger surface area, which can capture the analyte more efficiently; (ii) they can act as part of the recognition element; (iii) they utilize enzymes, aptamers, DNA, RNA, etc., as platforms for fixing biological recognition components; and (iv) the nanomaterial itself can act as a converter or amplifier for the recognition component signal.^84^ These sensors are portable and rapid, have a low cost and competitive performance qualification compared to traditional methods, and exhibit advantages for use on large numbers of samples in situ.^85,86^

### Importance of Coumarin Detection

Although coumarin is well absorbed from the gastrointestinal tract when administered, clearance times vary between species and range from 1 to 2 h in humans and from 1 to 4 h in other creatures. Degradation of coumarin leads to the creation of active metabolites with therapeutic activity, and these molecules are supposed to be prodrugs. Coumarin and its derivatives have a wide range of bioactive features, including antioxidant, anticoagulant, antibacterial, anti-inflammatory, antitumor, antiviral, and enzyme inhibitory effects. It is also helpful in mitigating the risk of cancer and other brain and cardiovascular system diseases. Although these effects are mainly attributed to the impact of free radical scavengers, they are hepatotoxic in high doses.^52^ Some coumarin compounds act as phytoalexins that rapidly accumulate at sites of parasite infection because they have antiparasitic features. Psoralen, found in citrus fruits, is a furanocoumarin with antiarthritic, antibacterial, and anti-inflammatory effects. Apart from this, esculetin, another coumarin has been reported to protect single-cell DNA from oxidative attack and inhibit aldose reductase activity associated with diabetic neuropathy, nephropathy, and retinopathy.^87,88^ In addition, simple coumarin has aromatic properties and is used in cosmetics such as sunscreen. The fluorescence of coumarins is used in several biochemical methods; after absorbing one particular wavelength, they emit light of another wavelength. In living cells, simple coumarins are used to study the enzymatic movement and pharmacokinetics of the corresponding medicine. Because they are light-sensitive, they absorb ultraviolet rays and have a blue fluorescent color.^89^ Coumarin has antitumor and bacteriostatic effects and may even be used to cure diseases such as brucellosis caused by the consumption of raw dairy products. They contain immunomodulators that help strengthen the immune system. Therefore, coumarins have great potential as future medicines but have yet to be tested in clinical trials.^42^ As we mentioned before, a slightly excessive dose of coumarin can cause toxic effects on human and animal bodies. Even though it is poisonous, it continues to be used in food. Detection of coumarin is important because it is both toxic to living things and beneficial to the scientific community. For these reasons, scientists have accelerated studies on the detection of coumarin, especially in recent years.^90,91^

Coumarin detection may also find a place in agriculture. Agricultural output is directly related to the rhizobacterial communities, which respond to the type of coumarins the plant synthesizes.^92^ This is partly due to the direct antimicrobial effect of coumarins against certain groups of bacteria.^93^ Another reason for the impact of coumarins on bacterial communities arises from their ability to bind iron.^94^ While iron is an essential nutrient for plant growth, it is crucial for the emergence of infections in all living things, including plants.^95^ Therefore, recent advancements in understanding the role of coumarins in plant–microbe interactions and plant nutrition underline their importance.

## Sensors

### Basic Information about Sensors

Sensors are devices that acquire the composition, structure, and function of molecules by transforming biological reactions into electrical impulses.^96^ Sensors connect the sensing element to a physical transducer like an electrochemical, optical, or piezoelectric transducer to convert the interaction between the target and sensor molecules into an evaluable electrical impulse.^97^ Sensor systems provide fast, accurate, and label-free detection, decrease analysis time, and require necessary steps for sample preprocessing. Thus, these systems offer solid alternatives to traditional analytical methods.^98^ In addition, sensor systems are integrated into various sciences, such as chemistry, nanotechnology, physics, biology, and electronics. This integration has developed the work of existing sensor systems in terms of analysis time, sensitivity, application, remote monitoring capability, and usability. Recognition has been improved with molecularly imprinted polymers (MIPs) that provide better recognition and stability, thereby reducing the limitations of antibody–protein systems.^99^ Molecularly imprinted polymer-based sensors have been improved for scanning targets in many platforms, such as medical diagnostics, food pollution, and environmental protection.^100−103^

For instance, electrochemical sensors are more familiar than other types of sensors due to their essential features including cost-effectiveness, size, and portability.^104−106^ These sensors are typical sensing platforms incorporating semiconductors and screen-printed electrodes. In short, electrochemical sensors monitor any changes in dielectric properties, size, shape, and charge distribution during the creation of an antibody–antigen complex at the electrode surface. They can be divided into four main groups: potentiometers, cyclic voltammeters, amperometers, and impedance converters. The portability and cost-effectiveness of electrochemical sensors allow them to be used as devices to provide medical care to patients at home or in the clinic, making electrochemical sensors appropriate candidates for sensing uses.^107,108^ Quartz crystal microbalance (QCM) sensors, a type of piezoelectric sensors, measure changes in the mass and viscoelasticity of materials by recording changes in the frequency and damping of the quartz resonator. They are a type of analytical device that operates on the piezoelectric principle.^109^ They have attracted the interest of scientists thanks to their features, including stability, portability, and high specificity. QCM sensors allow for the observation of interactions between oscillating crystals and biomolecules immobilized on their surface. The coupling response occurs as associated with the increase in mass, resulting in a decrease in vibration rate. Due to its high sensitivity to environmental conditions, the detection mechanism requires isolation equipment that minimizes interfering factors such as vibration.^108^ Combining QCM sensors with memory template molecules through prerecognition with MIPs enables more sensitive sensing systems depending on template affinity, highly selective binding sites, and homogeneity across more significant recognition sites.^110^ In optical sensors, recognition and target elements form a complex. Therefore, the sensors mentioned focus on measuring changes in the optical properties of the transducer surface. In optical sensors, which are divided into direct and indirect optical sensors, signal generation in direct optical sensors depends on developing a complex on the transducer surface. On the other hand, indirect optical sensors are adorned with an array of tags, such as fluorophores or chromophores, to determine coupling events and increase the signal. Various optical sensors, such as optrode-based fibers, transient wave fibers, time-resolved fluorescence sensors, resonance mirrors, interferometrics, and surface plasmon resonance sensors, are available in the literature and are used in many fields. The versatile detection window enables the sensing of many molecules in physiological and biological samples.^111^

Table 1 describes the general advantages and disadvantages of the main sensors.

**Table 1:**
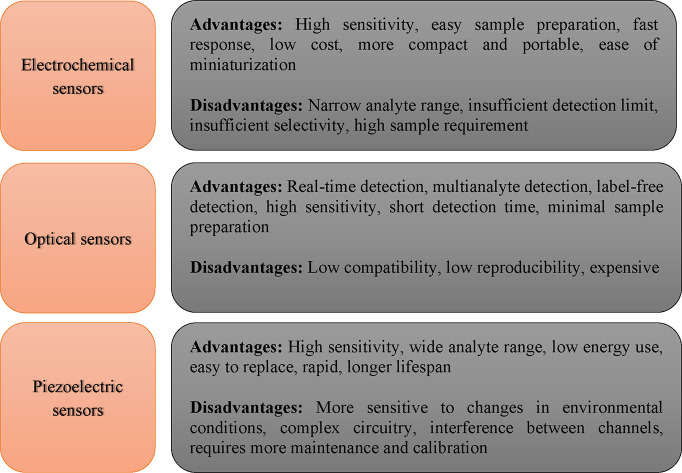
General Advantages and Disadvantages of the Sensors

### Importance of Sensors for Detection

**Figure 2:**
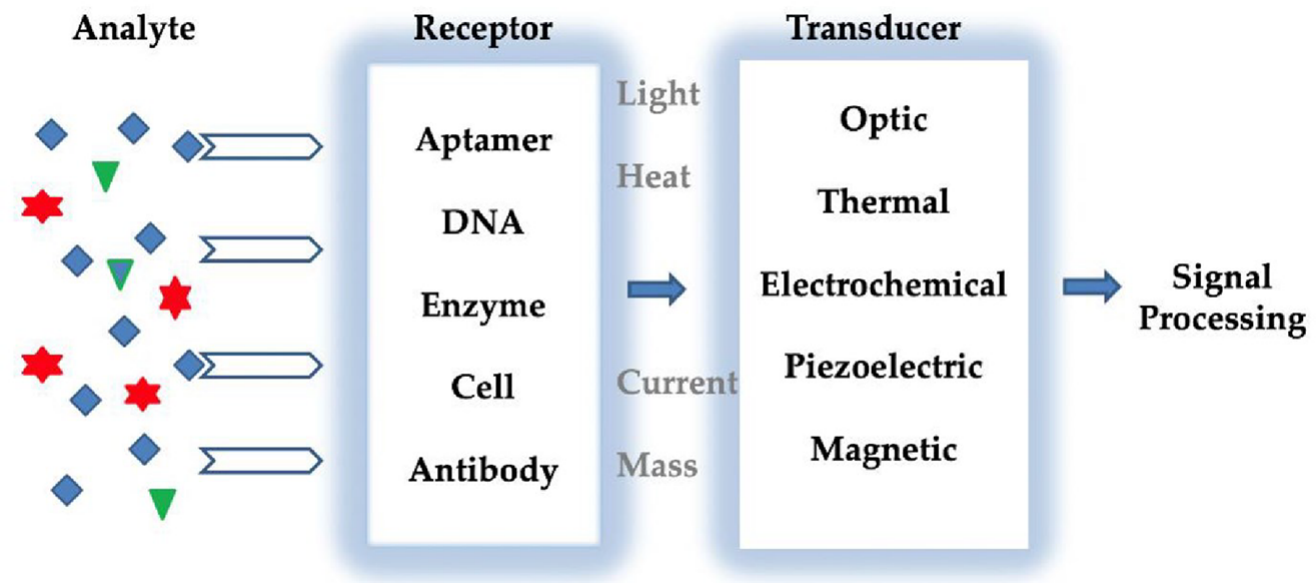
Typical detection principle of a sensor. Reprinted with permission from ref (128). Copyright 2022 MDPI.

The detection of biological components is necessary in several areas, including food processing,^9,23−25,104^ clinical medicine,^8,26,112,113^ environmental control,^5−7,72,81,83,114,115^ and healthcare.^11,22,27,105,116,117^ Therefore, there is a great demand to develop safe and economical devices for a healthy lifestyle. The sensor is a crucial device studied to detect various gas molecules and biomolecules.^118^ Sensors transform physical, chemical, and biological variations in the environment into electrical signals.^119,120^ Typically, sensors have essential parts: transducers, electronic components, and receptors (Figure 2). The basis of perception depends on the particular dynamic between the analyte and the receptor.^121−123^ Depending on the interaction, transducers detect properties, including changes in the pH, temperature, electrons, mass transfer, optical properties, and potential. The system transforms the receptor response into an electronic signal directly related to the presence of the analyte or corresponding to the concentration of the analyte. Analytes used in sensing applications are described as analytes and structures to be analyzed. Receptors are elements that are part of compounds or mixtures. Although enzymes and antibodies are more well-known, dyes, polymers, and chelating agents are also used as receptors to regulate the sensor surface. Transducers are parts of sensors that transform the detected energy from one form to another. The selectivity of the sensors is a critical parameter, as they must respond to analytes in complex matrices of actual samples.^124^ Optical, piezoelectric, and electrochemical transducers are exploited in sensors to measure and transmit the signals resulting from the interplay of samples and ligands. The mentioned sensor technologies are commonly used in industries such as pharmacology, environmental analysis, biomedical, and healthcare.^125−128^

## Nanomaterials

Nanomaterials have opened up new aspects of science and technology and created new possibilities in various biological applications such as sensing, anticancer therapy, separation, and molecular imaging. All biological and artificial systems have first-order organization at the nanoscale, where their essential properties and functions are defined. Nanotechnology provides tools and technological platforms to study and modify biological systems, and biology provides inspiring models and biologically assembled components for nanotechnology. These properties have spurred the discovery of new materials’ unknown physical and chemical properties by precisely controlling their structure and composition for specific technological applications.^129^ Several related materials have been reported to be applied in sensors.^130−132^ Conducting polymers have exceptional electron donor or acceptor properties that make them perfect for sensing applications. As a result, conductive polymers have been used to provide sensor properties.^133^ Nanocarbon nanostructures, especially graphene and carbon nanotubes, are used in sensing applications.^134^ Among the conducting polymers, polyaniline is often chosen for optical sensors.^135^

### Types of Nanomaterials

Nanomaterials are divided into two groups, natural and artificial nanoparticles, according to their origin.^136^ Natural nanomaterials are present in nature in many forms, such as viruses, protein molecules, minerals like clay, natural colloids like milk and blood, fog, gelatin, mineralized natural materials such as corals, shells and bones, insect wings, opals, gecko feet, spider silk, lotus leaves, volcanic ash, and ocean spray. Semiconducting nanoparticles including carbon nanotubes and quantum dots are artificial nanomaterials intentionally created using mechanical and manufacturing methods. Nanomaterials are divided into metal-based materials, dendrimers, or composites according to their structural composition.^137,138^

**Figure 3:**
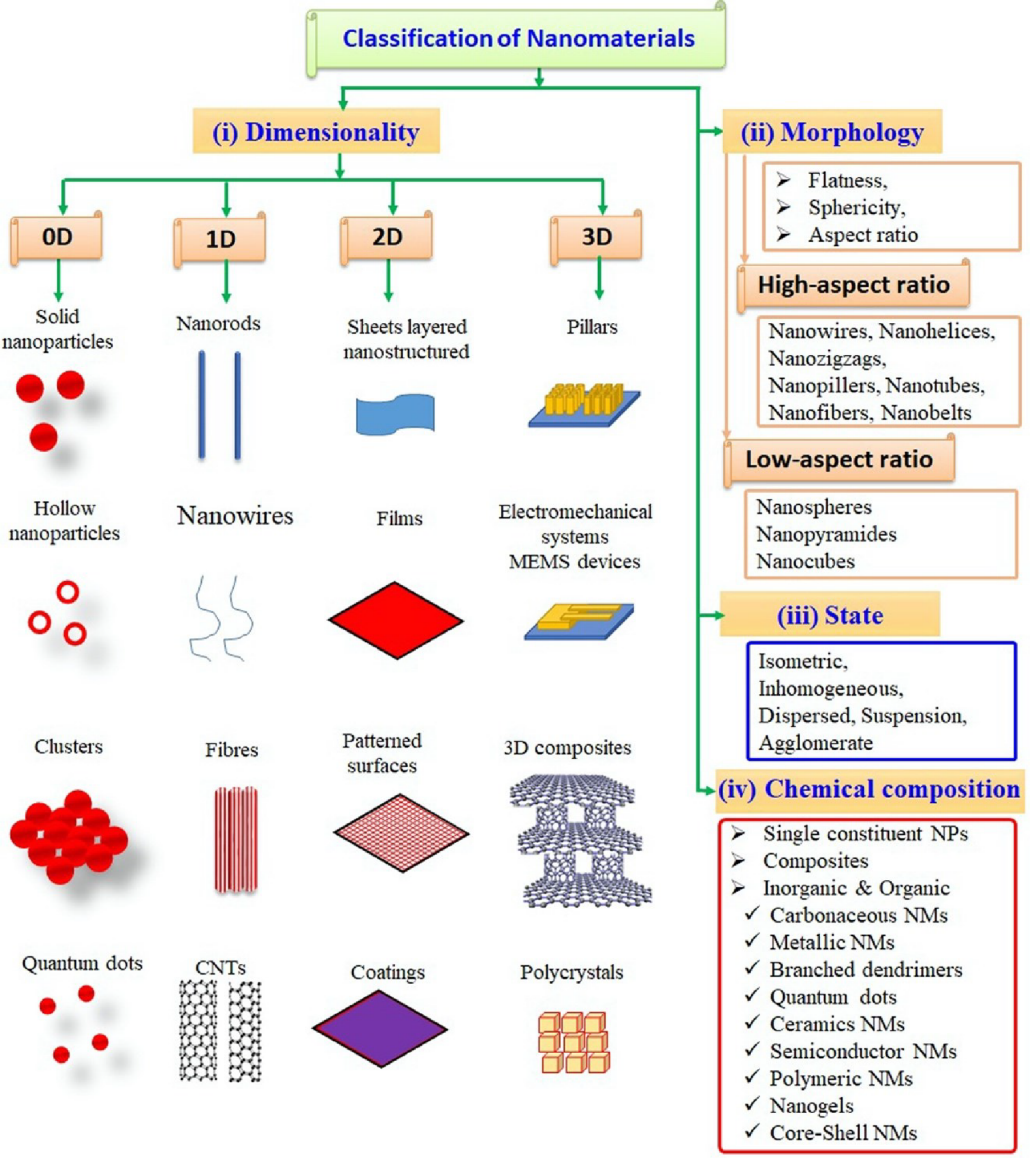
Classification of nanomaterials. Reprinted with permission from ref (142). Copyright 2020 Elsevier.

**Figure 4:**
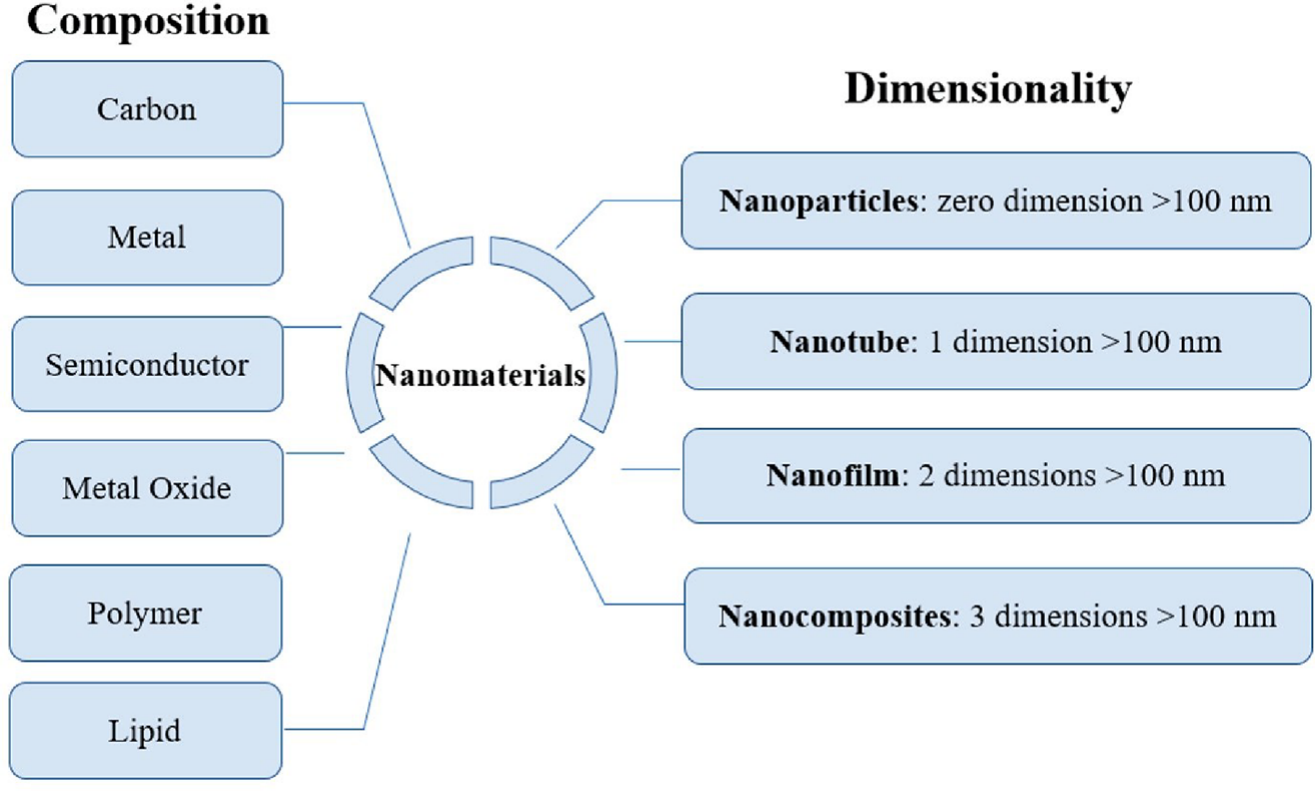
Classification of nanomaterials depends on the composition and dimensionality. Reprinted with permission from ref (150). Copyright 2021 MDPI.

Depending on their size and shape, nanomaterials can be divided into four categories.^139−142^ Zero-dimensional (0D) nanomaterials have all dimensions on the nanoscale, that is, less than 100 nm in size. Spheres, hollow spheres, cubes, nanorods, polygons, metals, core–shell nanomaterials, and quantum dots are 0D nanomaterials. Polymeric materials, ceramics, nanotubes, nanorods, nanowires, and nanofibers are all one-dimensional (1D) nanomaterials with only one non-nanometer dimension. Materials consisting of only one nanoscale length, including monolayer and multilayer types, nanofilms, nanoplates, nanocoatings, and crystalline or amorphous types, are two-dimensional (2D) nanomaterials. As depicted in Figure 3, three-dimensional (3D) materials have dimensions measuring greater than 100 nm and combine multiple nanocrystals in different directions, such as foams, fibers, layer skeletons, carbon nanobuds, nanotubes, fullerenes, pillars, polycrystals, and honeycombs.^143,144^ In addition, the combination of different nanomaterials has led to the emergence of new components called nanocomposites. Nanocomposites combine different properties of different materials to provide improved or new physical and chemical properties. Therefore, nanocomposites play an important role in the development of sensors.^145^ The best sensors should also have a rapid response time, low production cost, long service life, and small size. Today, the integration of 0D, 1D, 2D, and 3D nanomaterials and their nanocomposites represents a prominent research area in the development of sensors.^146−149^Figure 4 depicts the classification of nanomaterials depending on their composition and dimensionality.

#### Metallic Manoparticles

Nanoparticles are nanoscopic particles ranging in size from 1 to 100 nm, consisting of materials such as carbon, metals, metal oxides, and polymers.^151−154^ Compared to bulk materials, nanoscale materials can change their physicochemical, mechanical, and biological properties to a considerable extent. Nanoparticles have many advantages, such as improved bioavailability and long residence time due to their small size and surface functionality.^155^ In some literature, they are also called nanoclusters, which usually consist of up to 100 atoms and have appropriate physicochemical properties. The word “nanoparticle” is derived from the Greek word “nano” meaning “dwarf” or “small”, and when used as a prefix it indicates that 10^–9^ billionths of a meter in size equals 1 nm. Nanoparticles have both solution and phase properties of the individual particles. Nanoparticles have 35–45% higher surface area ratios than large particles or atoms. This unique extrinsic property of nanoparticle-specific surface area contributes to their high cost and affects various intrinsic properties such as the strong size-dependent surface reactivity. These exceptional properties of nanoparticles are generally responsible for their multifunctional properties and increase the interest in their applications in various platforms such as energy, medicine, and nutrition. Currently, the synthesis of metal nanoparticles (MNPs), nanostructures, and nanomaterials has attracted attention from researchers due to the excellent properties of the materials. These properties are advantageous for composite-like polymer preparations, catalysis, disease diagnosis and treatment, and sensor technology. MNPs consist of at least one metal element and can be obtained in various forms. Many of them have properties that differ from bulk metals due to their greater catalytic activity, large surface-to-volume ratio, and distinctive electromagnetic behavior.^156^ People come into direct contact with various metal particles in products, including cosmetics, soaps, detergents, toothpaste, shampoos, pharmaceuticals, and medicines. Gold nanoparticles (AuNPs) are commonly used in medicine in India and China, such as diagnostic and drug delivery applications. Additionally, other MNPs like silver nanoparticles (AgNPs) are also used for various biomedical applications such as separation science and new drug delivery systems. Silver, known for its antimicrobial and anti-inflammatory effects, supports wound healing. Thanks to this feature, it is used in wound dressings, various pharmaceutical dosage forms, and medical implant coatings. Like other nanomaterials, silver nanoparticles are used in various biomedical applications such as separation science and drug delivery systems, aiding in the treatment of wounds and injuries, various pharmaceutical dosage forms, and medical implantation.^157^

MNPs offer multiple advantages for biomedical use, such as strength, optical properties, elasticity, and electrical conductivity. These benefit their applications in tissue engineering, imaging and sensing, photothermal therapy, and other fields.^158,159^ Noble metal, magnetic metal (including iron, manganese, and cobalt), and metal oxide nanoparticles are important for various biomedical platforms like diagnostic and therapeutic use.^160^ In the area of theranostics, using MNPs in simultaneous treatment and diagnosis is an efficient approach. The unique properties, of MNPs, including a broad range of optical properties, slightly easy synthetic strategies, and various surface functionalization possibilities, present significant uses in biophysical, diagnostic, and therapeutic approaches. Two steps were taken to increase the functionality and compatibility of MNPs: the addition of stabilizers to prevent the aggregation of MNPs and the integration of MNPs into nanocomposites. These applications have led to the emergence of new structures that are more stable, have larger loading areas, and have better optical features and biological productivity.^161^

#### Nanofilm

Two-dimensional (2D) materials have received widespread attention thanks to their great mechanical, optical, electrical, and thermal features. Due to their excellent physical and chemical features, 2D materials are widely used in fields such as high-performance bionics, filtering, energy, and flexible electronic devices. 2D materials are often assembled in several layers to make nanofilms for practical use, which is important in various fields is due to challenges in the large-area preparation of single-layered 2D materials, their inherent structural instability, and the presence of numerous flaws. The performance and stability of composites or bendable electronic tools made from nanofilms are linked to the mechanical features of nanofilms. Utilizing nanofilms as the recruitment stage may also considerably boost the force and fracture restraint of composite materials.^162^

Nanofilm biomaterials are “nanoscopically” thin polymer-based functional films that serve as biocompatible interfaces. As a result, films including carbon nanotubes have been shown to have powerful antimicrobial features and are therefore promising materials for biomedical devices that are inherently resistant to microbial infection. Studies are ongoing to build films with independently controllable mechanical force and biological activity.^163^ Nanofilms are ultrathin layers of material with a thickness ranging from fractions of a nanometer to a couple of micrometers. They represent the atomic thickness boundary with the environment at which most of the physicochemical procedures happen. Hence, the existence of a thin layer of a particular material can impact the activity and provide the capability to divide volumetric and surface design. Stacks of oppositely charged multilayers are possibly the largest class of nanofilms, where the density of states is limited to 2D regulation and quantum matching between multilayers determines the features of the multilayer. Layer-by-layer self-assembly is a widely used technique for fabricating multilayered thin films. Various approaches are available for the deposition of individual layers: dip-coating, spin-coating, sputtering, electromagnetic deposition, and liquid–liquid deposition.^164^

#### Carbon-Based Nanomaterials

Improvement of carbon-based nanomaterials as antiviral agents after appropriate modification or interaction with fitting polymers could improve systems and increase compatibility and therapeutic efficacy. Additionally, carbon-based nanomaterials have high a specific surface area, which allows them to be functionalized or interact with appropriate biocompatible or bioactive materials such as chitosan, alginate, and cellulose, thereby enhancing target properties and biosafety.^165^ As a result of research conducted during the pandemic, carbon-based nanomaterials have shown possible antiviral activity against severe acute respiratory syndrome coronavirus 2 (SARS-CoV-2) with versatility and low toxicity. Because of their distinctive chemical structure, these nanomaterials may help develop targeted drug delivery systems for SARS-CoV-2 without inducing an immune response in reticuloendothelial cells. Wide surface areas aid in enhancing the drug transport ability. They can also pass through negatively charged films by changing the intrinsic charge of the surface.^166^ Properties of carbon nanotubes including flexibility, low density, and high mechanical strength make them appropriate for sensing and blocking certain viruses. These nanomaterials have stability against most acids and bases. Carbon-based nanomaterials are often used to strengthen structures because they are sometimes tougher than steel. Although carbon-based nanomaterials are thermally conductive along their entire length, they do not conduct heat through the tubes.^167,168^

Graphene and its derivatives are among the most researched members of carbon-based nanomaterials. Graphene is a single-atom-thick carbon layer on the sp^2^ carbon structure and has good electrical conductivity. Graphene has a large surface area that relies on a single layer of carbon atoms to detect individual molecules. Sensors are created by attaching biomolecules to the graphene surface. Their ability to interact with light and absorb visible light plays an important role in heat production. Additionally, the use of reduced graphene oxide can provide a highly hydrophilic surface for adsorbing nucleic acids and proteins and exhibit stronger antibacterial and antiviral activity.^169^ Carbon nanomaterials have fullerenes and nanotubes as major subclasses.^170^ Fullerene, a zero-dimensional nanomaterial, has antiradical and antioxidant features.^171^ Because of the high hydrophobicity of the starting fullerenes, it is possible to synthesize antiviral derivatives of fullerenes to obtain hydrophilic drugs that dissipate effortlessly in aqueous media.^172^ Due to their wide electroactive surface area, fullerenes are frequently utilized to create electrochemical sensors to sense amino acids and DNA.^173,174^ Small spherical fullerenes are carbon nanoparticles that effectively interact with pathogens and cells. Carbon nanotubes have hollow and round designs. They can be used as sensors to detect various elements, such as immunoglobulins. The needle-like structure of nanotubes allows them to penetrate cells to treat diseases.^175^ Depending on the needs and modifications, carbon nanotubes can behave like semiconductors or superconductors.^176^ Carbon dots (CDs) are living assemblies of fluorescent carbon nanomaterials with small crystalline graphitized cores and polymeric surface groups.^177−179^ Due to their structure, CDs have unique optical and electronic properties,^180−182^ such as very high fluorescence quantum yields, great stability, red/near-infrared emission, and dispersion properties.^183−185^ It is not surprising that compact discs are increasingly used in the production of electronics, power conversion devices, and sensors.^186−188^ Moreover, owing to their eccentric optical, physical, and chemical properties, CDs and CD-based nanomaterials carry the biggest potential for various biological and biomedical uses, such as for imaging and therapy.^189^

The metal oxides are the most complete class of materials in terms of optoelectronic, magnetic, electrical, photoelectrochemical, thermal, electrochemical, mechanical, and catalytic properties. This diversity is due to the more complex crystal and electronic structures of metal oxides compared to other materials. Metal oxide-based sensors possess attractive features such as low detection limits, cost-effectiveness, great sensitivity, and ease of use. Metal oxide semiconductor sensors are mainly used to detect toxic exhaust and flammable gases.^190^ Chemically stable semiconductor metal oxides are good candidates for gas detection for their properties including cost-effectiveness, fast response and recovery times, plain electronic interface, ease of use, and ability to detect a variety of gases.^191^

#### Other Types of Nanomaterials

Semiconductor nanoparticles consist of semiconductor materials, and their properties are between those of metals and nonmetals. Compared with bulk semiconductor materials, these nanoparticles have a wide band gap and exhibit important changes in their features with the adjustment of the band gap. Therefore, semiconductor nanoparticles are significant materials in photocatalysis, electronics, and optical tools.^140^ Semiconductor nanomaterials consist of many compounds from different families, and by changing the structure of these materials at the nanoscale it is possible to change the chemical and physical features of the material because of the quantum size effects or increased surface area.^192^ Semiconductor nanomaterials have metallic and nonmetallic properties.^193^ There are two types of semiconductor nanomaterials. Intrinsic semiconductors are pure compounds that are not doped with other metals in the structure. Extrinsic semiconductors are materials added to other metals through doping to improve their conductivity, such as n-type and p-type semiconductors.^194^

Solid lipid nanomaterials are used in drug delivery. These nanomaterials have some useful features including chemical and physical stability, cost-effectiveness, site targeting, the ability to supervise hydrophilic and hydrophobic molecules, and nontoxicity.^195^ In addition, they also have negative properties: since these materials crystallize during storage, they may show limited drug loading and mobility. Nanostructure lipid carriers are intended to be used as materials for controlled drug release and offer stability compared to solid lipid nanomaterials.^196^ Liposomes are 50–100 nm in size and are formed by cholesterol compounds and phospholipids. They are appropriate for carrying drugs such as cytotoxic medicine due to their decreased toxicity and increased bioavailability.^197^

Ceramic nanomaterials have been found to have improved electro-optical, structural, superconducting, ferroelectric, and ferromagnetic features. Ceramics are a combination of metallic and nonmetallic elements, often oxides, phosphates, nitrides, and carbides. There is a wide variety of ceramic materials, such as clay minerals, cement, and glass, which are used for various purposes. These materials are usually electrical and thermal insulators and are highly resistant to aggressive chemical environments compared to metals and polymers. Regarding mechanical behavior, ceramics are very hard and brittle.^198,199^ Polymeric nanomaterials are composed of natural or synthetic materials in the form of nanometer-sized solid particles.^200^ These materials are frequently used as drug release controllers for body detection in medical and pharmaceutical applications. Polymeric nanomaterials include polymeric micelles, polymeric nanoparticles, dendrimers, and polymeric nanocomposites. Polymeric micelles originated from the self-assembly of amphiphilic block copolymers in a particular solvent. Polymeric nanoparticles are typically prepared from biodegradable and biocompatible polymers in the size range of 10–1000 nm. Dendrimers smaller than 15 nm have three-dimensional macromolecules. Polymeric nanocomposites formed by other nanofillers and polymers deliver excellent features and performance.^201^

### Importance of Nanomaterial-Based Sensing

Nanomaterials are ideal candidates for such sensing arrays because they are easy to fabricate, are chemically versatile, and can be integrated into currently available sensing platforms. Nanomaterials have unique physical and chemical properties such as size dependence, quantum confinement, high specific surface area, and excellent catalytic activity. These distinctive features give the sensor high sensitivity, reliability, and a rapid response time. With the emergence of the Internet of Things (IoT), the need to increase sensor manufacturing has emerged, which has stimulated intense research on nanomaterial-based sensors. Additive manufacturing of nanomaterial-based sensors is important to bridge the gap between one-off laboratory-scale production and cost-effective highly reproducible industrial-scale production. Thanks to the design flexibility and cost savings inherent in additive manufacturing technology, next-generation nanomaterial-based sensor platforms can be integrated with IoT devices in the consumer sector.^202^

The application of nanoscale sensors in the field of food safety has developed the ability to detect foodborne pathogenic microorganisms, drug residues, toxic contaminants, and pesticides in agricultural products that threaten human health. The presence of nanomaterials in the structure of sensor technology has led to the development of more advanced devices that are easy to use, highly sensitive, and provide better detection rates. Furthermore, it provides sensors capable of detecting individual analytes of toxic chemical or biological contaminants in the food sample. In addition, having biomolecules for biorecognition of antigen–antibody interactions provides the possibility of improving the detection specificity or selectivity of food-borne pathogens or analytes.^203^ Diagnosing multifactorial diseases is challenging due to the lack of suitable sensors that can detect low concentrations of molecularly imprinted polymers and peptides and create signals that can be measured using electricity through biochemical interactions. Sensors based on nanomaterials are the most popular and reliable tools in this context, achieving both goals at a relatively low cost while being robust and portable. These sensors are mainly fabricated on various nanomaterials, such as graphene, quantum dots, carbon nanotubes, magnetic nanomaterials, nanofibers, and some imprinted structures. These new application aspects bring new horizons to materials science research on small sensing elements, especially the detection of elements that no other physically feasible sensing element can achieve. The use of sensors ranges from environmental protection by detection of pesticides and water pollution to the detection of drug residues in food and drinking water. Sensors are currently used in forensics because of their intracellular detection capabilities. Sub-micrometer laser sensors can detect organisms in living cells. Obviously, the ultimate goal is to develop laboratories-on-a-chip that will revolutionize the current healthcare system and be accessible to all financial classes.^204^

## Limitations of Nanomaterial-Based Sensors for Coumarin Detection

Although nanomaterial-based sensors have great potential for coumarin detection, some limitations need to be considered. First of all, it is necessary to strictly control the reproducibility and stability of nanomaterials and establish an efficient production process for sensors based on nanomaterials. In addition, to improve detection accuracy, most sensors use cDNA and aptamers as biological recognition elements so they have separate detectable objects, which limits their application in coumarin detection. Similarly, there are many types of samples, such as organic molecules, biomacromolecules, and other particles, that may interfere with the sensing process because they are easily adsorbed on the surface of nanomaterials, which will significantly affect the selectivity and stability of biorecognition molecules in sensors. Although nanomaterials are extremely sensitive, they tend to degrade in air, resulting in a short lifespan for the sensors. As a result, their development and practical application are further limited. Most importantly, since complex concentrations in real sample solutions greatly affect the analysis of nanomaterial-based sensors, pretreatment is necessary to remove these unwanted molecules before analysis. This limits the use of nanomaterial-based sensors in real sample analysis. Sensors based on nanomaterials are also difficult to prepare at standard scale, and these limitations still need to be removed.^97,205^

## Applications of Nanomaterial-Based Sensors for Coumarin Detection

**Figure 5:**
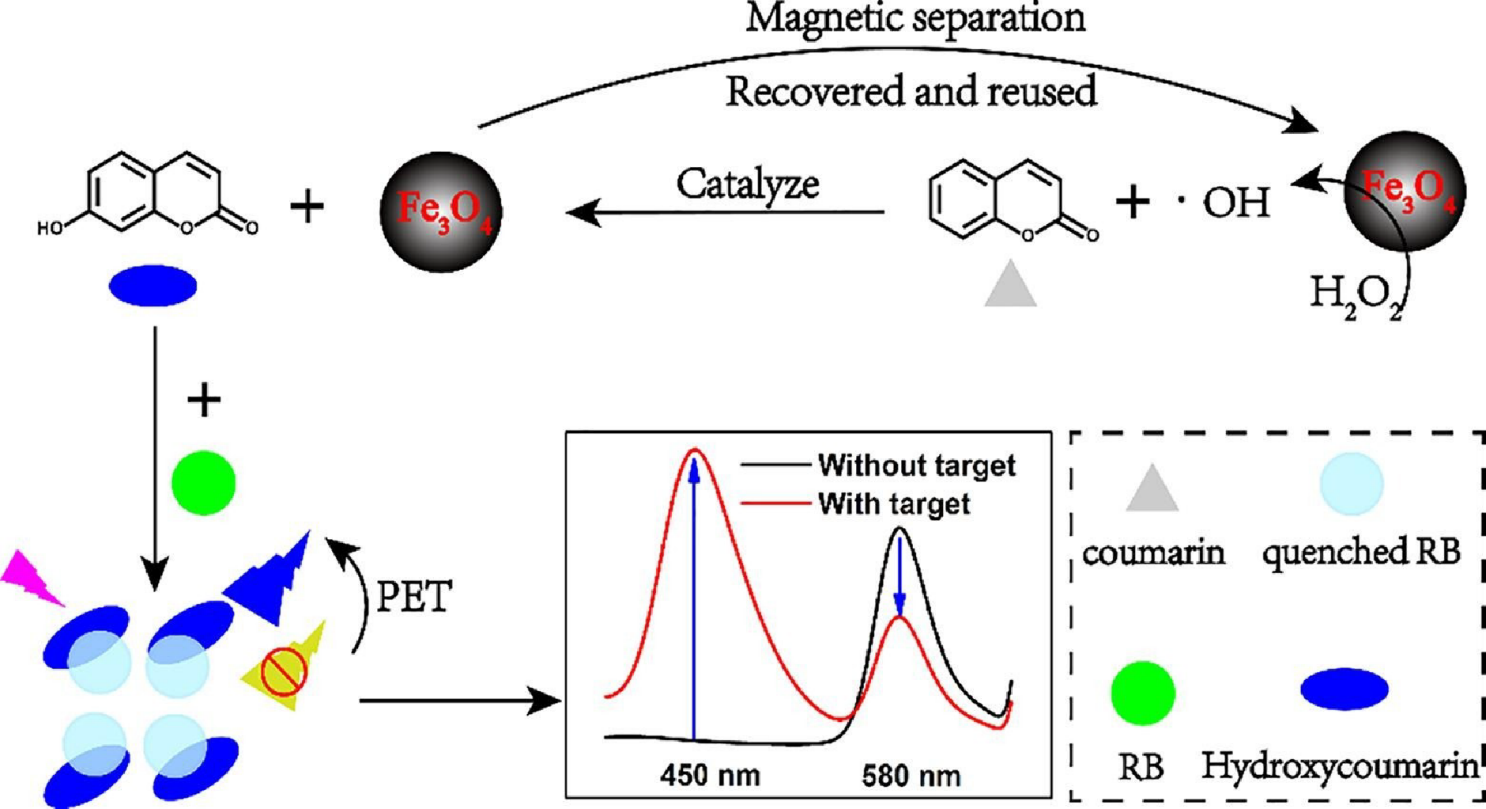
Schematic diagram of a photoluminescence sensor. Reprinted with permission from ref (206). Copyright 2022 Elsevier.

The latest applications of different types of nanomaterial-based sensors for coumarin detection are mentioned in this section. For instance, Yang et al. used metal nanoparticles (Fe_3_O_4_ NPs) to create a photoinduced electron transfer (PET)-based coumarin switch between 7-hydroxycoumarin and rhodamine B (RB) as a magnetic artificial peroxidase. The results show that the NPs catalyze H_2_O_2_ to attack the active site of coumarin, forming the nucleophilic groups and producing highly fluorescent 7-hydroxycoumarin molecules (Figure 5). The fluorescence of RB was then quenched by 7-hydroxycoumarin via PET impact. The generated rate signal was used for the quantitative detection of coumarin. Under optimized circumstances, the linear range of coumarin is 0.5–25 mg/L, and the limit of detection (LOD) of 0.016 mg/L.^206^

Fan et al. reported a ratiometric fluorescence sensor for detecting ultrasensitive glutathione (GSH) by modulating the oxidase-like properties of the MnO_2_ nanosheet (MnO_2_ NS). Unlike previous studies, MnO_2_ NS strongly suppressed the fluorescence of scopoletin (SC) and increased the fluorescence of Amplex Red (AR). When MnO_2_ NS is premixed with GSH, it is reduced to manganese ions (Mn^2+^) and loses its oxidase-like properties, which is followed by an increase in SC’s fluorescence and a decrease in AR’s fluorescence. The LOD of GSH is 6.7 nM.^207^ Yao et al. also designed a ratiometric fluorescence sensor for the detection of organophosphorus pesticides (OPs) using SC and Amplex Red (AR) as a probe pair with opposing reactions to MnO_2_ NS. Dichlorvos (DDVP) was selected as the model organophosphorus pesticide and, in the absence of AChE, could hydrolyze ATCh to acetate and choline. TCh induced the dissociation of MnO_2_ NS into Mn^2+^, enhancing SC signaling and decreasing AR signaling. It was observed that in the presence of OP the activity of AChE was inhibited and the decomposition of MnO_2_ NS was prevented. Thus, the fluorescence intensity of SC was low and the fluorescence intensity of AR showed a highly significant increase. The method has a wider linear range of 5.0 pg/mL to 500 ng/mL with a LOD of 1.6 pg/mL.^208^ Yang et al. presented a fluorescent sensor for coumarin detection based on fluorescence resonance energy transfer (FRET). β-Cyclodextrin (CD) was modified on NaYF and used to achieve selectivity toward coumarin and bring the donor and receptor into FRET proximity via a specific host–guest interaction. The linear response range is 2.5–32.5 μM, and the LOD is as low as 0.74 μM.^209^ Li et al. synthesized water-soluble and environmentally friendly nitrogen-doped graphene quantum dots (NGQDs) via a hydrothermal method. In the study, an optical sensor based on NGQDs (NGQDs@MIPs) coated with molecularly imprinted polymers via sol–gel polymerization was prepared. Demonstrating specific selectivity and superior detection performance for warfarin, the fluorescence intensity of the NGQDs@MIPs sensor presented an excellent linear response with warfarin concentrations ranging from 0.63 to 10 μM with a LOD of 0.16 μM.^210^

**Figure 6:**
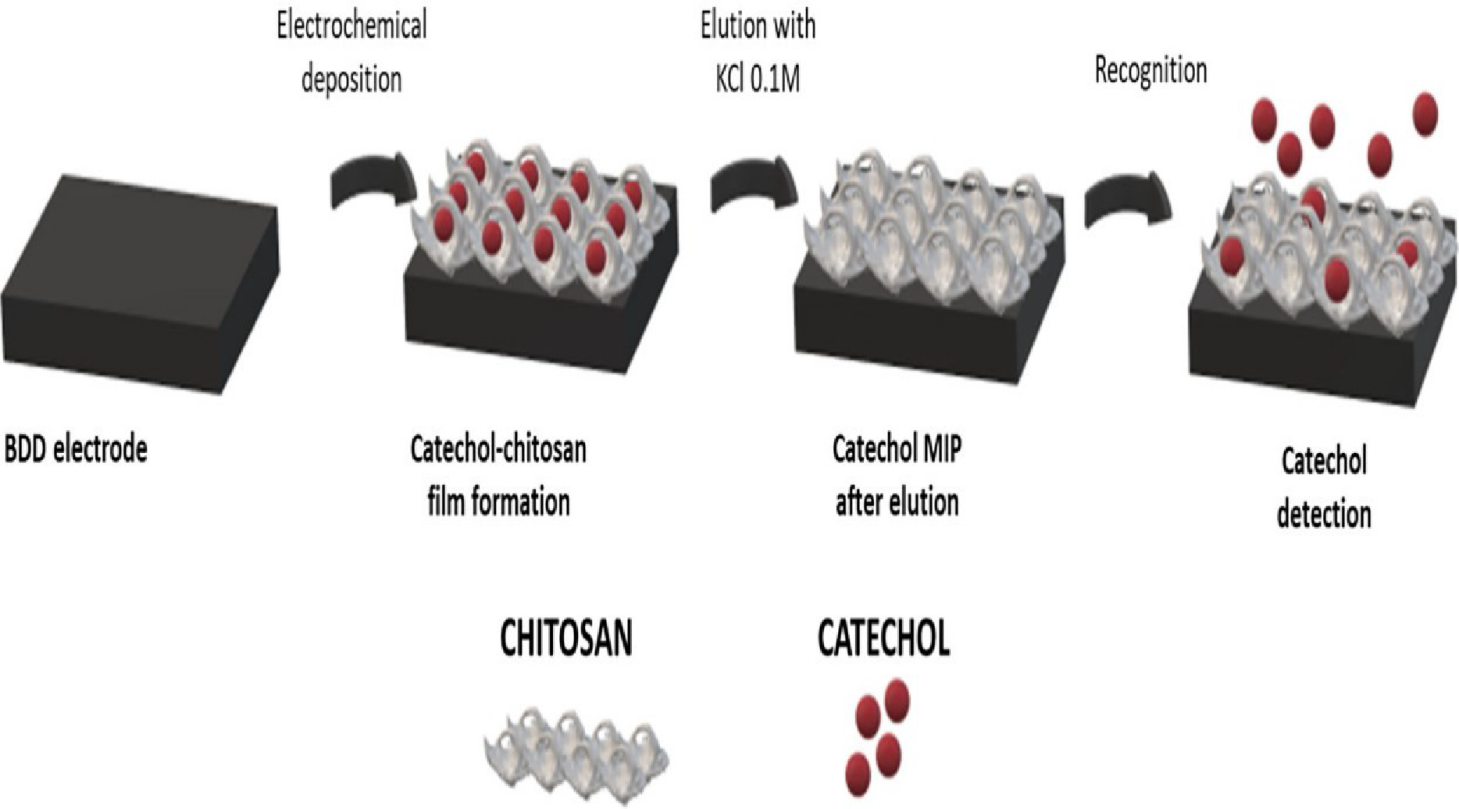
Schematic illustration of a voltammetric sensor for catechol detection. Reprinted with permission from ref (218). Copyright 2020 Elsevier.

Zhao et al. formed a molecularly imprinted electrochemical sensor for coumarin detection and applied AuNP-modified reduced graphene oxide (rGO) on a glassy carbon electrode (GCE). Modification of AuNP/rGO is carried out through the deposition of coumarin template molecules. The linear range and LOD of the sensor for coumarin detection were from 1.0 × 10^–7^ to 1.8 × 10^–5^ M and 1.15 × 10^–8^ M, respectively. The sensor has the characteristics of high sensitivity, good stability, and fast response speed and is awaiting utilization in real sample detection.^211^ Fu et al. proposed a method based on electrochemical reduction behavior, as detection of coumarins in samples requires an extraction that is too complex to meet the detection needs of the analytical technique. The sample particles were combined with graphene and immobilized on the electrode surface. After the preliminary reduction, the quantity of coumarin in the real sample can be assessed by voltammetry. The applicability of this method was analyzed using fruits and it was found to be successful.^212^ Huang et al. reported a coumarin detection method using surface-enhanced Raman spectroscopy (SERS) paired with smart multivariate analysis. First, it was characterized by producing a flower-like silver-based substrate, and then different concentrations of coumarins were detected using this substrate as a SERS substrate. The LOD of coumarin with a flower-like silver substrate can be as high as 10^–8^ M, meaning that it is less than 1.46 μg/kg.^37^ Yue et al. collected AgNPs designed by the molten salt method on the surface of hexagonal boron nitride nanosheets (Ns/AgNPs) from a composite. The synthesized nanocomposite was used to modify the surface of the screen-printing electrode (SPE) and successfully detect scopoletin. The electrochemical behavior of the Ns/AgNp/SPE was observed. The response of the presented electrochemical sensing platform was linear over a wide detection range from 2 to 0.45 μM with a low LOD of 0.89 μM.^213^ Sheng et al. developed the electropolymerization of l-lanthionine on a GCE. A voltammetric sensor based on l-lanthione was created. The electrochemical properties of poly(l-lanthionine)/GCE were studied by cyclic voltammetry and differential pulse voltammetry to measure esculetin. The sensor had a great sensitivity of 539.8 μA/mM cm^2^ in the 5.0–100 nM esculetin concentration range with a LOD of 1.0 nM.^214^ Zheng et al. build up a simple one-pot strategy to prepare flower-shaped yolk–shell SiO_2_ nanospheres (FYSSns) with a simple combination of cetyltrimethylammonium bromide–polyvinylpyrrolidone–cyclohexane–ethanol–aqueous vesicles–microemulsion composite pile via the solvothermal route. FYSSns exhibit a flower-shaped yellow shell structure (260–320 nm) with ordered radial mesochannels (∼7 nm), large voids (100–120 nm), and thin silica shells (20–30 nm). A high amount of laccase immobilization was achieved using FYSSns as a carrier, and laccase-modified FYSSns were coated onto an electrochemical sensor with great selectivity for catechol detection. Under optimized conditions, the proposed sensor exhibited a wide linear range of catechol concentration from 12.5 to 450 μM and a lower LOD of 1.6 μM.^215^ Zhang et al. developed a new laccase-based sensor for detecting catechol based on a nanocomposite of MoS_2_ NS and AuNPs. Results showed that MoS_2_ NS has a wide particular surface area and decent biocompatibility, delivering enough opportunity for enzyme immobilization. AuNPs increase the conductivity of MoS_2_ and increase the detection sensitivity. Due to the synergistic impact of MoS_2_ NS and AuNPs, the laccase-based bioelectrode showed decent selectivity, repeatability, stability, and reproducibility, along with a linear response to catechol from 2 to 2000 μM with a LOD of 2 μM.^216^ Salvo-Comino et al. reported the improvement of a biocompatible and sensitive MIP-based sensor for the electrochemical detection of catechol based on natural-biopolymer-electroactive nanocomposites. Multiwalled carbon nanotubes adorned with AuNPs were encapsulated in a chitosan polymer matrix, and this chitosan nanocomposite was used to prepare MIP on boron-doped electrodes in the presence of catechol. When the electrochemical response of the sensor was explored by cyclic voltammetry, great repeatability and reproducibility for catechol detection were observed in the range of 0–1 mM with a LOD of 3.7 × 10^–5^ M.^217^ In another study from the same year by Salvo-Comino et al., a chitosan MIP film was electrodeposited onto a boron-doped diamond electrode by chronoamperometry in the presence of catechol (Figure 6) and eluted with 0.1 M KCl. The morphology of MIP and non-MIP films was investigated using AFM. The electrochemical reaction of the sensor explored using cyclic voltammetry shows that the sensor exhibits great repeatability and reproducibility for the detection of catechols in the range of 0–80 μM along with a LOD of 6.9 × 10^–7^ M. Results acquired in red wine demonstrate that catechol can be detected in a complex matrix.^218^

**Figure 7:**
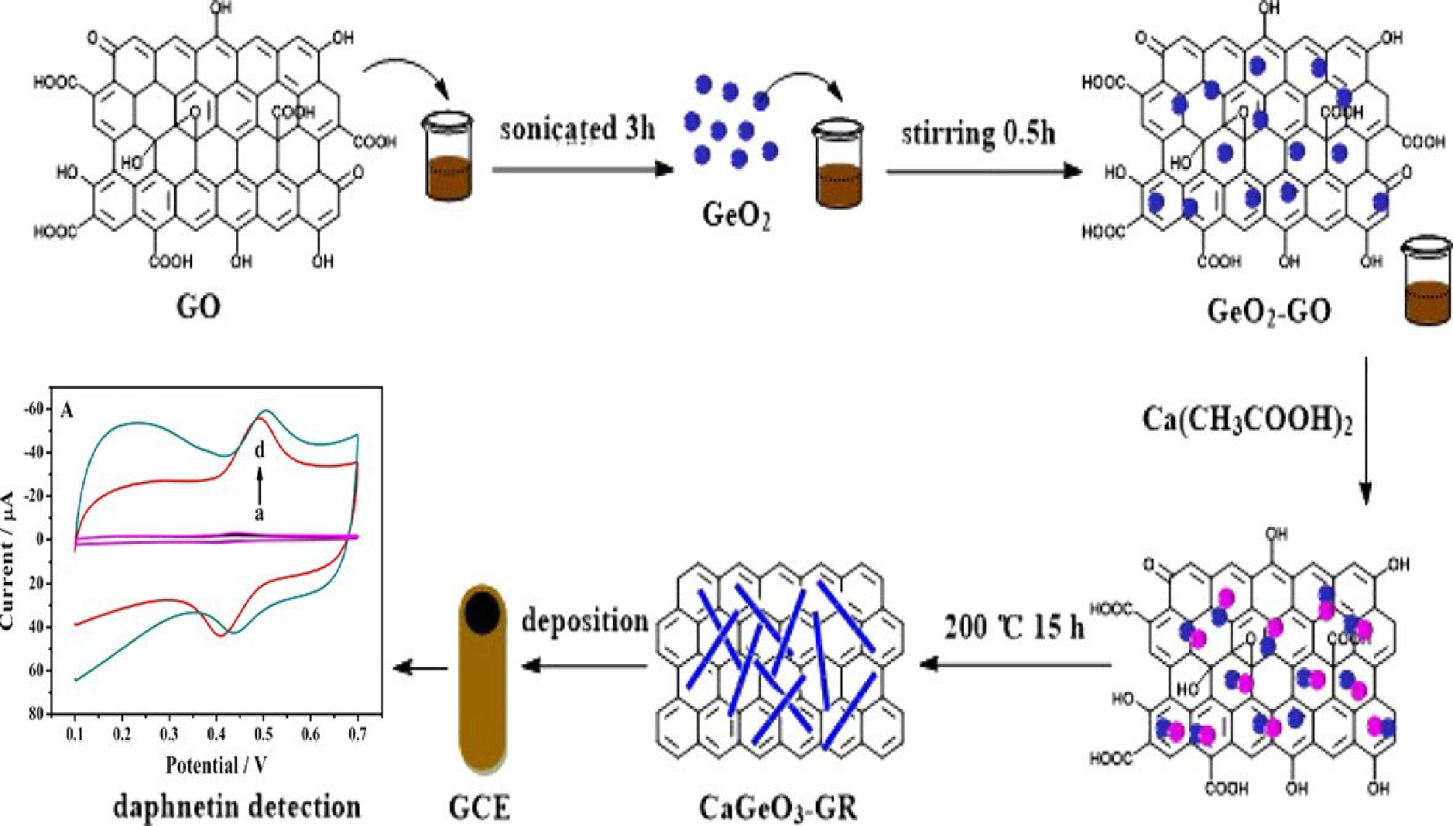
Schematic diagram of the electrochemical sensor for the determination of daphnetin. Reprinted with permission from ref (228). Copyright 2017 Elsevier.

Sandeep et al. described the improvement of a quite selective and sensitive electrochemical sensor for the detection of catechols by immobilizing crude polyphenol oxidase (PPO) enzyme on a graphite (Gr) electrode modified with graphene nanoribbons (GNRs) adorned with green synthesized AgNPs. The Gr/GNR/AgNPs/PPO electrochemical sensor developed was validated at each stage of its production by cyclic voltammetry and electrochemical impedance spectroscopy. The sensor developed in optimized states exhibited superior electrocatalytic activity for catechol detection. The sensor had a large detection range and a low LOD. The sensor even provided excellent selectivity in the detection of catechols in the case of joint interference.^219^ Yan et al. prepared sp-hybridized nitrogen atom-doped ultrathin graphdiyne (NUGDY) based on graphdiyne oxide and melamine through high-temperature carbonization. NUGDY retains the typical folded and wrinkled 2D morphology of GDY. It also has a 3D porous network structure that supplies enough interface and capacity to load target analytes. In this study, NUGDY modified the surface of the carbon ionic liquid electrode and used the modified electrode to analyze 6,7-dihydroxycoumarin. Differential pulse voltammetry studies demonstrate that the LOD of this electrochemical sensor is as low as 2.3 nM.^220^ Saeedi et al. presented a proof of concept of a new detection device based on ion-selective electrode technology for the direct detection of warfarin in blood samples without any sample pretreatment. Tetradodecylammonium chloride (TDDA) was used as an ion exchanger to produce an ion-selective membrane. The developed ion-selective electrode showed high sensitivity for detecting warfarin in buffer and blood, with LODs of 1.25 × 10^–7^ and 1.4 × 10^–5^ M, respectively. The sensor also showed promising selectivity in detecting the presence of various ions present in blood, with a calibration slope of 58.8 mV/dec.^221^ In recent years, electrochemical biosensors have become excellent tools for warfarin detection.^222−227^ Fu et al. developed an electrochemical daphnetin sensor using a nanocomposite of calcium germanate–graphene (Ca_2_GeO_4_-GR) as the electrode material (Figure 7). Here, Ca_2_GeO_4_ nanowires can be evenly distributed on the GR surface with an average diameter of approximately 30–60 nm. The prepared sensor shows a great current response to daphnetin with linear range from 2 × 10^–8^ to 9 × 10^–7^ mol/L and a LOD of 6 × 10^–9^ mol/L.^228^ Apart from this study, various electrochemical sensors have been used to detect daphnetin, including ERGO/GCE,^229^ SDS-GN/SnO_2_/GCE,^230^ and AuNPs-GH/GCE.^231^

Tokura et al. reported the ability to detect methylmercaptan (MM) at concentrations as low as 20 ppb using manganese oxide nanosheets coated with a QCM sensor. The sensing capability of manganese oxide nanosheets is supported by adsorbed water situated on and between the nanosheets. Strong adsorption of MM into the sensor is necessary for high sensitivity. This phenomenon causes a notable delay in the circuit response because of irreversible adsorption. However, heating to 80 °C can easily zero the sensor, providing highly reproducible responses to low MM vapor concentrations. The layered nanosheet fabrication of highly sensitive dilute MM sensors presented here holds great promise for detecting sulfur compounds important for environmental protection and medical diagnostics.^232^ Chi et al. developed a QCM sensor based on a layer of nitrogen-doped ordered mesoporous carbon-modified AuNPs (NOMC-Au) and cross-linked an imprinted layer of 3-thiophenoacetic acid-functionalized AuNPs (3-TAA@AuNPs) to detect acrylamide as a hazardous substance during thermal treatment. The composite modification layer formed by NOMC-Au was used as a support material for QCM gold chips (AuE). Then, 3-TAA@AuNPs were incorporated into the highly selective imprinting modification layer via electropolymerization using a propionamide as a model. The final QCM detection platform (MIP/NOMC-Au/AuE) showed a good linear range for acrylamide from 0.08 to 100 ng/mL. The recovery rate of the three additional concentrations of the three samples was between 88.3% and 97.2% and the LOD was 5.1 pg/mL.^233^

## Conclusions and Future Perspectives

The applications of nanoscale materials derived from carbon-based, metallic, or inorganic sources provide devices with greater cost effectiveness, low detection limits, long-term stability, high sensitivity, and selectivity. As shown, these nanoparticles can always form new nanocomposites with improved properties. They do this by easily combining with each other or other biological components. Their use also allows the miniaturization of the platform, allowing real-time monitoring of physiological parameters using portable devices. Additionally, miniaturization of the platform paves the way for integration with wearable electronics, which is an emerging field. This field represents one of the future directions in sensor device research and requires strong collaboration between different disciplines such as chemistry, engineering, materials science, and biology.

The selectivity and stability of sensor technology are crucial aspects that determine the effectiveness and reliability in various applications. Selectivity refers to a sensor’s ability to respond specifically to the target analyte or stimulus while ignoring interference from other substances or environmental factors. In other words, a highly selective sensor will detect only the intended target and not be influenced by irrelevant variables. Selectivity is typically achieved through the sensor’s design, including the use of specific recognition elements (such as antibodies, enzymes, or molecularly imprinted polymers) that interact only with the target analyte. Additionally, signal processing techniques may be employed to enhance selectivity by discriminating against interference signals. Moreover, stability in sensor technology refers to the sensor’s ability to maintain consistent performance over time and under varying environmental conditions, including factors such as temperature fluctuations, humidity, mechanical stress, and chemical exposure. A stable sensor will produce reliable and reproducible measurements over extended periods without significant drift or degradation of sensitivity. Achieving stability often involves the careful selection of materials for sensor construction, robust calibration procedures, and regular maintenance and quality control measures. Additionally, advancements in sensor packaging and encapsulation techniques help protect the sensing elements from external influences, thereby enhancing the stability. Thus, selectivity ensures that the sensor responds accurately to the target analyte, while stability ensures a consistent performance over time and under different conditions. Both characteristics are critical for the successful deployment of sensor technology in various fields, such as environmental monitoring, healthcare, industrial process control, and consumer electronics.

Interference in sensor technology refers to any external factor or condition that disrupts or distorts the accurate measurement or detection of the target analyte by the sensor. These interferences can arise from various sources and can significantly affect the reliability and performance of the sensor. For instance, cross-sensitivity occurs when a sensor responds not only to the target analyte but also to other substances present in the environment. This can lead to false readings or inaccurate measurements if the sensor cannot distinguish between the target analyte and the interfering substances. In addition, changes in environmental conditions, such as temperature, humidity, pressure, and electromagnetic interference, can influence sensor performance. Chemical substances present in the sample matrix or the surrounding environment can interfere with the sensor’s detection mechanism. This interference may result from chemical reactions, adsorption, or competing interactions with the sensing elements, leading to a reduced selectivity or sensitivity. Also, physical factors, such as mechanical stress, vibration, and shock, can impact sensor performance by causing damage to the sensor components or altering their mechanical properties. Physical interference can lead to sensor drift, calibration errors, and even sensor failure. In biological sensing applications, interference may arise from the presence of biological molecules, cells, or tissues that interact with the sensor’s recognition elements. Nonspecific binding or fouling of the sensor surface by biological substances can affect the sensor’s specificity and sensitivity. To mitigate this interference, sensor designers employ various strategies such as signal filtering, shielding, and calibration techniques and select robust materials and sensor configurations. Additionally, careful consideration of the operating environment and sample matrix characteristics is essential for minimizing interference and ensuring accurate sensor measurements.

The use of developed nanomaterials in sensing fields further expands their use in improving the responsiveness of designed sensors in photothermal states. The use of advanced nanomaterials to improve the quantum yield and antioxidant features further strengthens their role in biomedicine. The reusability and high response rate of the developed sensor further make it highly efficient in environmental reformation applications. Furthermore, nanomaterial-based sensors need to be combined with automated sample analysis and preprocessing systems. Currently, a small number of nanomaterial-based sensors have been obtained for integration into microfluidic systems. Lack of repeatability is a major problem in nanomaterial-based sensors due to the difficulty of placing nanomaterials on the sensor and controlling their structure. The manufacture and manipulation of nanomaterials are highly regulated and remain significant technical challenges. For this reason, high-level nanomaterials and their applications in sensing fields are very interesting areas of nanomaterial study. This has led to the emergence of a new field of research called nanostructuration, which is a conceptual paradigm for the design and synthesis of size-controlled functional nanomaterials. Another major obstacle is the high cost of large-scale production due to advanced processing and instrumentation. Therefore, another key area for future research is the development of low-cost and well-controlled fabrication processes for large-scale nanostructures in sensing devices. There are some potential issues associated with the detection of coumarins using nanomaterials. For instance, achieving a high sensitivity and selectivity can be challenging. Nanomaterials need to be engineered to selectively bind with coumarin, among various other substances. Other substances present in the sample can interfere with the detection process, leading to false positives or negatives. Ensuring that the nanomaterial-based detection method produces consistent and reproducible results can be difficult due to variations in nanomaterial synthesis and environmental conditions. Nanomaterials may degrade or change their properties over time, which can affect their performance in detecting coumarin. Producing nanomaterials with the required specifications can be expensive and challenging to scale up for widespread use. If the detection involves biological samples, then ensuring that the nanomaterials are biocompatible and do not induce any adverse effects is crucial.

The aim of this review was to highlight the advantages and mechanism of using a nanomaterial-based sensor for the detection of coumarins. It should be noted that nanomaterials with superior optical, luminescent, catalytic, magnetic, and electrical properties hold great promise in the field of sensing. The potential for new sensors to provide greater control over analytical performance lies in their ease of fabrication and the ability to tune the features of the different nanomaterials. The improvement of nanomaterials for sensing use is just in the beginning phase, and notable work is ongoing to develop well-marketed devices. The next generation of ultrasensitive sensors are still under development. Various nanomaterials are still unexplored in gas, chemical, and biological uses because of their wide range of applications. An example of this is the new application of biological sensing needed to detect infectious diseases and cancer. Nanomaterials are not advanced enough for use in different sensing platforms. Besides, the introduction of nanomaterials to innovative sensing platforms, such as plasmon-based sensors, is another important study area.

## Abbreviations

AChE: Acetylcholinesterase
AgNPs: Silver nanoparticles
AR: Amplex Red
ATCh: Acetylcholine chloride
AuNPs: Gold nanoparticles
AuNP/rGO: AuNP-modified reduced graphene oxide
Ca_2_GeO_4_^–^: Calcium germanate-graphene
CDs: Carbon dots
CE: Capillary electrophoresis
ELISA: Enzyme-linked immunosorbent assay
FRET: Fluorescence resonance energy transfer
FYSSns: Flower-shaped yolk–shell SiO_2_ nanospheres
GC: Gas chromatography
GC-MS: Gas chromatography–mass spectrometry
GCE: Glassy carbon electrode
GSH: Glutathione
HPLC: High-performance liquid chromatography
IoT: Internet of Things
LC-MS: Liquid chromatography–tandem mass spectrometry
LOD: Limit of detection
MIPs: Molecularly imprinted polymers
MM: Methylmercaptan
MnO_2_NS: MnO_2_ nanosheet
MNPs: Metal nanoparticles
NGQDs: Nitrogen-doped graphene quantum dots
NOMC-Au: Nitrogen-doped ordered mesoporous carbon-modified AuNPs
NS/AgNP: Hexagonal boron nitride nanosheet
NUGDY: Nitrogen atom-doped ultrathin graphdiyne
PET: Photoinduced electron transfer
QCM: Quartz crystal microbalance
RB: Rhodamine B
rGO: Reduced graphene oxide
SARS-CoV-2: Severe acute respiratory syndrome coronavirus 2
SERS: Surface-enhanced Raman spectroscopy
SC: Scopoletin
SPE: Screen-printing electrode
0D: Zero-dimensional
1D: One-dimensional
2D: Two-dimensional
3D: Three-dimensional
3-TAA@AuNPs: 3-Thiophenoacetic acid-functionalized AuNPs
